# An Optimised Live Attenuated Influenza Vaccine Ferret Efficacy Model Successfully Translates H1N1 Clinical Data

**DOI:** 10.3390/vaccines12111275

**Published:** 2024-11-13

**Authors:** Katarzyna E. Schewe, Shaun Cooper, Jonathan Crowe, Steffan Llewellyn, Lydia Ritter, Kathryn A. Ryan, Oliver Dibben

**Affiliations:** 1 Flu-BPD, BioPharmaceutical Development, R&D, AstraZeneca, Liverpool L24 9JW, UK; schewek@astrazeneca.com (K.E.S.); shaun.cooper@astrazeneca.com (S.C.); jonathan.crowe1@astrazeneca.com (J.C.); steffan.llewellyn@astrazeneca.com (S.L.); lydia.ritter@astrazeneca.com (L.R.); 2 UK Health Security Agency, Porton Down, Salisbury SP4 0JG, UK; kathryn.ryan@ukhsa.gov.uk

**Keywords:** influenza virus, live attenuated influenza vaccine, ferret, animal model, clinical translatability, vaccine efficacy

## Abstract

Between 2013 and 2016, the A/H1N1pdm09 component of the live attenuated influenza vaccine (LAIV) produced instances of lower-than-expected vaccine effectiveness. Standard pre-clinical ferret models, using a human-like vaccine dose and focusing on antigenic match to circulating wildtype (*wt*) strains, were unable to predict these fluctuations. By optimising the vaccine dose and utilising clinically relevant endpoints, we aimed to develop a ferret efficacy model able to reproduce clinical observations. Ferrets were intranasally vaccinated with 4 Log_10_ FFU/animal (1000-fold reduction compared to clinical dose) of seven historical LAIV formulations with known (19–90%) H1N1 vaccine efficacy or effectiveness (VE). Following homologous H1N1 *wt* virus challenge, protection was assessed based on primary endpoints of *wt* virus shedding in the upper respiratory tract and the development of fever. LAIV formulations with high (82–90%) H1N1 VE provided significant protection from *wt* challenge, while formulations with reduced (19–32%) VE tended not to provide significant protection. The strongest correlation observed was between reduction in *wt* shedding and VE (R^2^ = 0.75). Conversely, serum immunogenicity following vaccination was not a reliable indicator of protection (R^2^ = 0.37). This demonstrated that, by optimisation of the vaccine dose and the use of non-serological, clinically relevant protection endpoints, the ferret model could successfully translate clinical H1N1 LAIV VE data.

## 1. Introduction

Influenza virus infection in humans causes 3–5 million severe infections and 290,000–650,000 deaths a year, globally [1], resulting in considerable economic impact and burden to health care systems [2,3]. Vaccination remains the most effective means of preventing influenza.

Serum neutralising antibodies against the influenza virus haemagglutinin (HA) protein are considered the correlate of protection for influenza vaccines [4,5,6]. Influenza viruses rapidly evolve. The accumulation of mutations in the HA protein results in a process referred to as antigenic drift, which leads to the loss of cross-reactivity of anti-HA antibodies [7]. As a result, seasonal influenza vaccines are updated on an annual basis.

Each year, the World Health Organisation (WHO) selects reference strains that represent currently circulating influenza viruses for each subtype/lineage [8]. Candidate vaccine viruses (CVVs) developed for inclusion in commercial vaccines must be shown to be antigenically similar, or ‘matched,’ to the relevant WHO reference strain. This cross-reactivity is assessed by the haemagglutination inhibition (HAI) assay, using post-infection ferret antisera. To be considered matched, the HAI titres of the CVV antisera against the CVV itself and the wildtype (*wt*) reference strain must be <4-fold different [9].

In the 2013–2014 and 2015–2016 influenza seasons, the A/H1N1pdm09 component of the FluMist/Fluenz live attenuated influenza vaccine (LAIV) produced reduced vaccine effectiveness (VE) in its quadrivalent (QLAIV) formulation [10,11]. However, the A/H1N1pdm09 strains used commercially, A/California/07/2009 (A/CA09) in 2013–2014 and A/Bolivia/559/2013 (A/BOL13) in 2015–2016, were antigenically matched to WHO reference viruses. This raised the possibility that antigenic characterisation alone was not sufficient to predict the VE of the A/H1N1pdm09 LAIV strains.

An investigation into the root cause of reduced A/H1N1pdm09 VE revealed that the efficient replication of A/H1N1pdm09 LAIV strains in primary human nasal epithelial cells (hNEC) was critical [12]. Both A/CA09 and A/BOL13 produced reduced replication in hNEC, relative to a clinically highly effective H1N1 strain, A/New Caledonia/20/1999 (A/NC99). Despite its reduced hNEC replication, A/BOL13 was still found to be immunogenic and protective in monovalent (MLAIV) form in ferrets; only producing reduced efficacy in trivalent (TLAIV) or QLAIV formulations [13]. Comparatively, A/NC99 provided significantly improved protection when substituted for A/BOL13 in the 2015–2016 formulations. This demonstrated that vaccine virus replication and inter-strain competition in multivalent vaccines was a significant factor in A/H1N1pdm09 LAIV VE.

These studies relied on the use of clinically relevant endpoints as the primary measures of LAIV protection from *wt* infection, including *wt* virus shedding in the upper respiratory tract and the development of fever post-challenge. Traditional animal model endpoints such as measurement of the *wt* virus load in the lungs were found to be of limited use due to unreliable infection of the lower respiratory tract [13]. Importantly, it was also found that the vaccine dose had a marked effect on the ability to distinguish differences in protection due to competition [13]. A model derived from this work, using a 4.0 Log_10_ FFU/ferret QLAIV dose, was later applied to investigating the efficacy of a novel A/H1N1pdm09 LAIV strain, A/Slovenia/2903/2015 (A/SLOV15), optimised for enhanced hNEC replication [14]. This represented a dose reduction of approximately 1000-fold, compared to the routine antigenic characterisation of MLAIV CVVs.

As with any influenza vaccine, LAIV has produced a range of H1N1 vaccine effectiveness or efficacy outcomes over time. In randomised controlled clinical trials for TLAIV, A/NC99 produced multiple observations of high efficacy in children (>80%), relative to both placebo and inactivated influenza vaccine (IIV) [15,16,17]. While A/NC99 had been shown to provide superior protection to A/BOL13 in ferrets [13], such data had not been generated for clinically applied A/NC99 vaccine formulations.

Following the 2009 H1N1 pandemic, the prototypic A/CA09 strain was used commercially in MLAIV form during the pandemic itself, then in multiple TLAIV and QLAIV formulations between 2009 and 2015, producing VE estimates of between 19% and 86% [11,18]. This raised the possibility that vaccine composition might contribute to the VE of an individual A/H1N1pdm09 strain with reduced replication in hNEC. Subsequently, A/BOL13 provided a consolidated 2015–2016 H1N1 VE estimate of 32% across multiple real-world studies [11], while in 2017–2018, the A/SLOV15 strain, optimised for hNEC replication, produced 90% VE in the UK [19]. Although in vivo QLAIV efficacies for A/BOL13 and A/SLOV15 were described separately [13,14], no comprehensive demonstration of the ferret model’s ability to consistently reproduce varying H1N1 VE estimates has yet been performed.

Here, a series of seven LAIV formulations, representative of preparations used either in randomised controlled clinical trials or in real-world applications, each with known H1N1 effectiveness or efficacy (herein, collectively referred to as VE), were investigated. We aimed to show that the use of a ferret-optimised vaccine dose and clinically relevant endpoints could consistently reproduce H1N1 clinical observations. Secondly, we aimed to determine whether variable VE for a single strain with reduced replication in hNEC (A/CA09) could be explained by changes in vaccine composition across influenza seasons. Finally, we aimed to determine whether serum anti-HA antibody responses in ferrets correlated with protection for H1N1 LAIV in this model.

The availability of a model able to reliably translate clinical data in vivo would provide an invaluable tool for improving understanding of the mechanism of action and correlates of protection of LAIV, as well as for developing approaches for the rational optimisation of H1N1 LAIV CVVs.

## 2. Materials and Methods

### 2.1. Animals

Studies were conducted in outbred, mixed-sex (equal male:female), influenza-free, 14–26-week-old ferrets (*Mustela putorius furo*) at Charles River Laboratories (CRL) Ltd., Ballina, Ireland, or the United Kingdom Health Security Agency (UKHSA), Porton Down, UK. Ferrets were sourced from either CRL, Ballina, or from a UK Home Office-accredited supplier, Marshalls Biosciences, UK (UKHSA). Ferrets were confirmed as seronegative for circulating H1N1, H3N2, and B viruses by HAI assay and randomly assigned to study groups (equal male:female split).

### 2.2. Cells and Eggs

As detailed in previous publications [13,14], Madin–Darby canine kidney (MDCK) cells and human embryonic kidney (HEK)-293T cells were cultured and maintained in Eagle’s Minimum Essential Medium (EMEM) (BioWhittaker; Lonza, Basel, Switzerland; Cat. No. BE12-662F) containing non-essential amino acids and sodium pyruvate and supplemented with 10% (*v*/*v*) heat-inactivated foetal bovine serum (FBS) (Gibco; Thermo Fisher Scientific, Waltham, MA, USA; Cat. No. 10500056), 1% penicillin–streptomycin (*v*/*v*) (Gibco; Thermo Fisher Scientific, Waltham, MA, USA; Cat. No 15140122), and 1% 200 mM L-Glutamine (*v*/*v*) (Gibco; Thermo Fisher Scientific, Waltham, MA, USA; Cat. No. 25030018).

Specific pathogen-free embryonated hen’s eggs were obtained from CRL, Wilmington, USA. Eggs were incubated at 37.5 °C with rotation and 70% humidity for 10–11 days prior to being inoculated.

### 2.3. Vaccines and Viruses

All LAIV strains were produced by reverse genetics, as previously described [20,21]. Briefly, an 8-plasmid system, encoding the 6 ‘internal’ genes (PB2, PB1, PA, NP, M, NS) of either the A/Ann Arbor/6/60 or B/Ann Arbor/1/66 master donor viruses and the HA and neuraminidase (NA) genes of the required candidate vaccine virus, were transfected into HEK-293T/MDCK cell co-cultures. After 72 h, rescue supernatants were harvested and inoculated into embryonated hen’s eggs to expand.

The strain compositions of the 7 vaccine formulations used are detailed in Supplementary Table S1. Abbreviations for the H1N1 strains are included.

Wildtype (*wt*) challenge strains were homologous to the H1N1 LAIV strains used and were either egg-derived *wt* viruses, propagated in embryonated hen’s eggs (*wt* A/BOL13, *wt* A/NC99, *wt* A/CA09), or cell-derived *wt* viruses, propagated in MDCK cells (*wt* A/SLOV15). Use of a cell-derived *wt* virus for A/SLOV15 was necessary because egg-derived *wt* A/SLOV15 shed to reduced levels and failed to induce a fever response in ferrets post-challenge (Supplementary Figure S1), making it inappropriate for assessment of vaccine-induced protection. The HA and NA genes of all LAIV and *wt* viruses were sequenced by Sanger sequencing and confirmed to be identical to the relevant *wt* strain.

All LAIV and *wt* virus formulations were quantified by fluorescent focus assay (FFA), similarly to previous descriptions [13,14]. Serial 1:3 dilutions of vaccine formulations were prepared in EMEM (BioWhittaker; Lonza; Cat. No. BE12-662F) with 50 μg/mL gentamicin sulphate (Life Technologies, Carlsbad, CA, USA; Cat. No. 15750–078), 2 mM L-glutamine (Sigma, St Louis, MO, USA; Cat. No. 25030081), and 0.5 μg/mL amphotericin B (Life Technologies, Cat. No. 15290018). MDCK cells were infected with 100 μL of serial dilutions and incubated at 33 °C and 5% CO_2_ for 18–20 h in the absence of trypsin. Plates were fixed with 80% acetone (VWR, Cat. No.100033P) in water and stained with relevant strain-specific, polyclonal anti-HA protein primary antibodies, followed by alexa-488 donkey anti-sheep IgG (H&L) secondary antibody (Life Technologies, Carlsbad, CA, USA; Cat. No. A11015). Here, fluorescent foci were quantified using the Cytation 5 high content imager (Agilent Technologies Inc, Santa Clara, CA, USA) and vaccine virus concentration was calculated as Log_10_ FFU/mL.

### 2.4. Ferret Challenge Studies

Ferret efficacy study designs were based on previous publications [13,14,22]. Data loggers (CRL: ANIPILL 0.1C, Data Sciences International. UKHSA: DST nano-T, Star-Oddi) were surgically implanted into the peritoneal cavity 10–15 days prior to vaccination. The data loggers were programmed to collect temperatures hourly from implantation to study termination.

On day 0 of each study, four ferrets per group were lightly sedated with isoflurane and intranasally vaccinated with a 0.2 mL dose (0.1 mL/nare) containing 4.0 Log_10_ FFU/strain of the appropriate LAIV formulation or a mock vaccination with sample diluent only (PBS with 1× sucrose phosphate, ThermoFisher Scientific, Waltham, MA, USA: custom product, Cat. No. AC10210390, and 1× gelatine–arginine–glutamate, ThermoFisher Scientific, Waltham, MA, USA: custom product, Cat. No. AC10207676).

Nasal swab samples (CRL) were collected daily on study days 1–5 by anaesthetising the animals with an intramuscular injection of 0.1 mg/kg Medetor (Medetomidine; Chanelle Veterinary, Loughrea, Ireland; Cat. No. PH003) and then sedating with isoflurane. A Copan FloqSwab (Copan, MINI (UTM Universal Transport Medium)) kit 1 mL (medium plus perinasal flocked swab, Cat. No. 360C) was inserted and rotated in the right nostril, then eluted by light vortexing in 1 mL of Copan universal transport medium before being aliquoted and stored at –80 °C until subsequent measurement of virus shedding. The anaesthetic was then reversed with an intramuscular injection of 0.1 mg/kg Revertor (Chanelle Veterinary, Cat. No. PH005), a minimum of 30 min after the sedative. Nasal wash samples (UKHSA) were collected daily on study days 1–5. Ferrets were sedated with isoflurane and nares were washed using 2 mL of PBS (Gibco). Nasal washes were collected and stored at –80 °C until subsequent measurement of virus shedding.

On day 21 post-vaccination, 2 mL bleeds were collected for the analysis of serum immune responses.

On day 28 post-vaccination, ferrets were lightly sedated with isoflurane as above, before being inoculated with 5.0 Log_10_ FFU/dose of *wt* challenge virus as a 0.2 mL dose (0.1 mL/nare). Nasal swab or nasal wash samples were then collected daily, for 3 days post *wt* challenge to allow measurement of *wt* shedding by TCID_50_ assay. Ferrets were euthanised three days post-challenge by intracardiac injection of an anaesthetic overdose (sodium pentobarbitone [Dolethal]; Vetquinol UK; 140 mg/kg). Nasal turbinates (NT) were collected into RNAprotect (Qiagen, Hilden, Germany; catalog no. 76163).

### 2.5. Quantification of Fever

Temperatures from intraperitoneal data loggers were analysed as described previously [13,14]. Briefly, for each *wt* challenge virus, post-*wt* challenge temperature profiles of unvaccinated control group animals were used to define a ‘fever period,’ the window during which the average body temperature was >1.5 standard deviations above the average pre-challenge baseline body temperature. Delta-temperature (body temperature vs. baseline) values were then calculated for all animals in the study by subtracting their baseline body temperature from each of the temperature values that were obtained during the fever period. A single ‘fever’ value was then derived by calculating the mean delta-temperature value during the fever period.

### 2.6. WT Virus Quantification by TCID_50_

Infectious virus titres were measured by the 50% tissue culture infectious dose (TCID_50_) in MDCK cells, and expressed as Log_10_ TCID_50_/mL, as previously described [14,23]. Ten-fold dilution series of virus containing samples were prepared in EMEM (BioWhittaker; Lonza, Basel, Switzerland; Cat. No. BE12-662F) with 1:400 10×TrypLE (Gibco; Life Technologies, Carlsbad, CA, USA; Cat. No. A1217701). MDCK cells were inoculated into 96-well tissue culture plates with the dilution series and incubated at 33 °C and 5% CO_2_ for 6 days. To score infection, 50 μL per well of 40 μM 2′-(4-Methylumbelliferyl)-α-D-N-acetylneuraminic acid sodium salt hydrate (MUNANA: Sigma-Aldrich, St Louis, MO, USA; Cat. No. M8639-25MG) in PBS was added per well and incubated for 1 h at 37 °C followed by 1 h at room temperature. The reaction was stopped by the addition of 50 μL of stop solution (0.1 M glycine, pH 10.7, with 25% ethanol). Fluorescence was immediately measured using a SpectraMax M5 plate reader (Molecular Devices), with 355 nm excitation and 450 nm emission. Virus-positive wells were scored as those with fluorescence readings ≥2-fold higher than the mean of uninfected control wells. TCID_50_ titres were then determined by the Spearman–Karber method.

### 2.7. Serum Immune Responses: HAI

HAI assays were performed as described in previous studies. In total, 100 μL ferret antiserum was combined with 150 μL 2× receptor destroying enzyme (RDE—Deben Diagnostics, Cat. No. 370013) and incubated at 37 °C for 18–20 h. In total, 150 μL 2% (*w*/*v*) sodium citrate was then added followed by heat inactivation at 56 °C for 45 min. Treated antisera were diluted in PBS if necessary (e.g., for high-titre MLAIV antisera). Antisera were 2-fold serially diluted and 8 HAU of virus was added to each well. Plates were incubated at room temperature for 30–40 min before the addition of 0.5% chicken or turkey red blood cell suspension (Envigo, Horst, Netherlands). Plates were incubated for a further 60 min and HAI titres recorded as the reciprocal of the highest dilution of antiserum able to fully prevent agglutination.

### 2.8. Analyses and Statistics

To facilitate analysis of *wt* virus shedding data, a single statistic of ‘shedding per day’ was calculated, to account for changes in shedding over time. This was taken as the geometric mean of daily shedding data points for each individual animal.

Statistical comparison of multiple groups was performed by ANOVA with Tukey’s multiple comparison test. Statistical significance was reached at an adjusted *p* value of 0.05. For comparison of two groups, statistical comparison was made by Student’s *t*-test. In the figures, statistical significance is represented as follows: * *p* < 0.05, ** *p* < 0.01, *** *p* < 0.001, **** *p* < 0.0001.

Relationships between endpoints and published VE (%) values were assessed by linear regression, calculated based on the group median values. The coefficient of determination (R^2^) and *p* values, calculated by ANOVA, are presented for each plot.

All statistical analyses were conducted in Graphpad Prism, version 9.

## 3. Results

**Figure 1:**
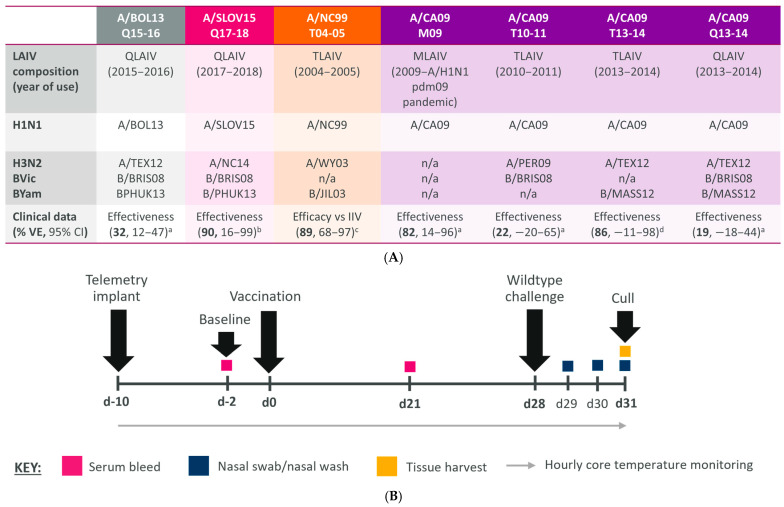
LAIV formulations with known H1N1 efficacy/effectiveness were assessed in an optimised ferret efficacy model. (**A**) Table detailing seven LAIV formulations used clinically between 2004 and 2018 with known H1N1 efficacy/effectiveness, assessed for their ability to protect ferrets from homologous *wt* H1N1 challenge. The H1N1 component is highlighted. H3N2 and B virus strains are also shown (strain abbreviations are detailed in Table S1). H1N1 efficacy/effectiveness data were taken from [11] ^a^, [19] ^b^, [15] ^c^, [18] ^d^. (**B**) Study schedule for the vaccination and challenge of ferrets. The timeline shows study days, with vaccination occurring at d0 and *wt* challenge at d28. Coloured boxes and black arrows indicate interventions/sampling as shown in the key or in text labels.

To assess the in vivo efficacy of the H1N1 LAIV strains used clinically between 2004 and 2018, seven LAIV formulations with known VE were selected (Figure 1A). QLAIV formulations containing A/BOL13 (2015–2016 QLAIV) and A/SLOV15 (2017–2018 QLAIV) were described in ferrets previously [13,14]. Here, these formulations were re-assessed by a different institution, using ferrets from a distinct colony, to help demonstrate the reproducibility and robustness of the model. Five additional formulations covering a range of VE estimates were then selected. The pre-2009 H1N1 strain A/NC99 provided a high efficacy (89%, 95% CI, 68–97) in a TLAIV formulation vs. IIV in a randomised controlled clinical trial in the 2004–2005 season [15]. Four A/CA09 vaccine formulations with differing VE were then selected from between 2009 and 2014: A/CA09 pandemic MLAIV (A/CA09 M09), USA (82%, 95% CI, 14–96) [11]; 2010–2011 TLAIV (A/CA09 T10-11), USA (22%, 95% CI, −20–65) [11]; 2013–2014 TLAIV (A/CA09 T13-14), Canada (86%, 95% CI, −11–98) [18]; and 2013–2014 QLAIV (A/CA09 Q13-14), USA (19%, 95% CI, −18–44) [11]. Each A/CA09 vaccine contained distinct H3N2 and B virus compositions (Figure 1A, and in full in Supplementary Table S1).

The study schedule for the vaccination and challenge of ferrets is shown in Figure 1B. In brief, 10–15 days prior to vaccination, mixed-sex ferrets (*mustela putorious furo*) of approximately 16–26 weeks of age received intraperitoneal implants of data loggers. These tracked core body temperature for the analysis of fever development as a measure of influenza-like illness. Baseline bleeds were taken at day −2 (d−2) to confirm seronegativity to currently circulating influenza viruses.

At d0, groups of four ferrets were intranasally vaccinated with 0.2 mL (0.1 mL/nare) of either ‘mock’ vaccine (vaccine vehicle) for unvaccinated control groups or 4 Log_10_ FFU/strain of a single LAIV formulation. Each LAIV formulation was administered to four animals, except for A/CA09 Q13-14 and its associated mock vaccine control, which was administered to eight animals separated across two studies one year apart, to further incorporate variation into the model. Based on previous evidence that LAIV virus shedding at a low LAIV dose did not predict post-challenge outcomes [13], LAIV virus shedding was not measured here. A serum bleed was then taken at d21 for measurement of H1N1 serum immunogenicity by HAI assay. At d28, each ferret group was challenged with 5 Log_10_ FFU of the *wt* H1N1 virus homologous to the vaccinating H1N1 LAIV strain. Post-challenge, nasal swabs or washes were taken daily for three days, followed by a cull and harvest of nasal turbinate (NT) tissues at d31. These samples were used to quantify *wt* virus shedding along with the *wt* virus load in upper respiratory tract tissues.

### 3.1. H1N1 Serum Immunogenicity Does Not Correlate with VE

**Figure 2:**
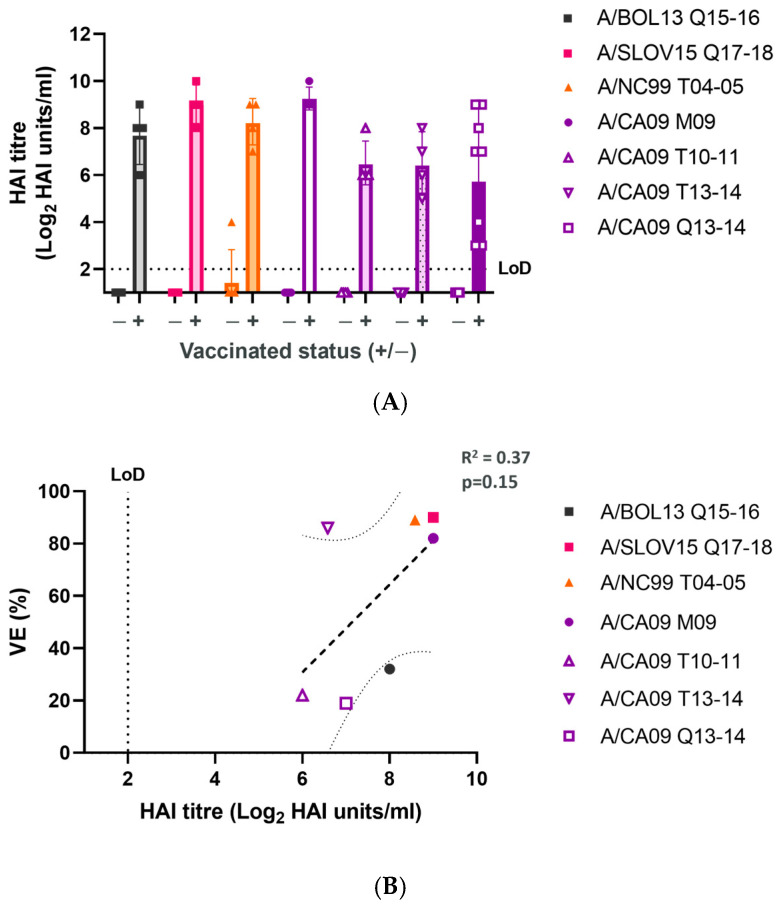
H1N1 serum immune responses provide limited differentiation of LAIV formulations with varying VE. (**A**) Serum HAI titres for unvaccinated and vaccinated animals. Points represent the Log_2_ HAI titre for individual animals. Columns and error bars show the group geometric mean titre (GMT) and geometric standard deviation. The dashed horizontal line represents the limit of detection (LoD) of the assay. Values below the LoD are arbitrarily shown as ½ LoD. (**B**) Linear regression comparing group GMT (coloured points) with VE. The linear regression line (heavy dashed line) and 95% confidence intervals (light dotted lines) are shown. Linear regression statistics (R^2^, *p* value) are shown.

The serum immunogenicity of H1N1 LAIV strains was measured at d21 post-vaccination (Figure 2). Using the vaccinating H1N1 LAIV virus as the target antigen for each group, serum HAI titres were generated (Figure 2A). All unvaccinated groups remained seronegative, while the highest group geometric mean HAI titres (GMT) were observed for A/SLOV15 Q17-18, A/NC99 T04-05, and A/CA09 M09. However, the range between the highest and lowest GMTs was approximately 3 Log_2_ (8-fold), limiting the ability to distinguish vaccine groups by this endpoint. Comparison of GMT by ANOVA with Tukey’s multiple comparison test showed no significant differences between any of the vaccinated groups. While a trend for increased GMT correlating with increased VE was evident (Figure 2B), this relationship was relatively weak (R^2^ = 0.37) and was not statistically significant (*p* = 0.15). These data suggested that serum immunogenicity in ferrets does not provide a useful indication of H1N1 LAIV VE.

### 3.2. LAIV Formulations with Higher H1N1 VE Provide Superior Protection from wt Challenge

**Figure 3:**
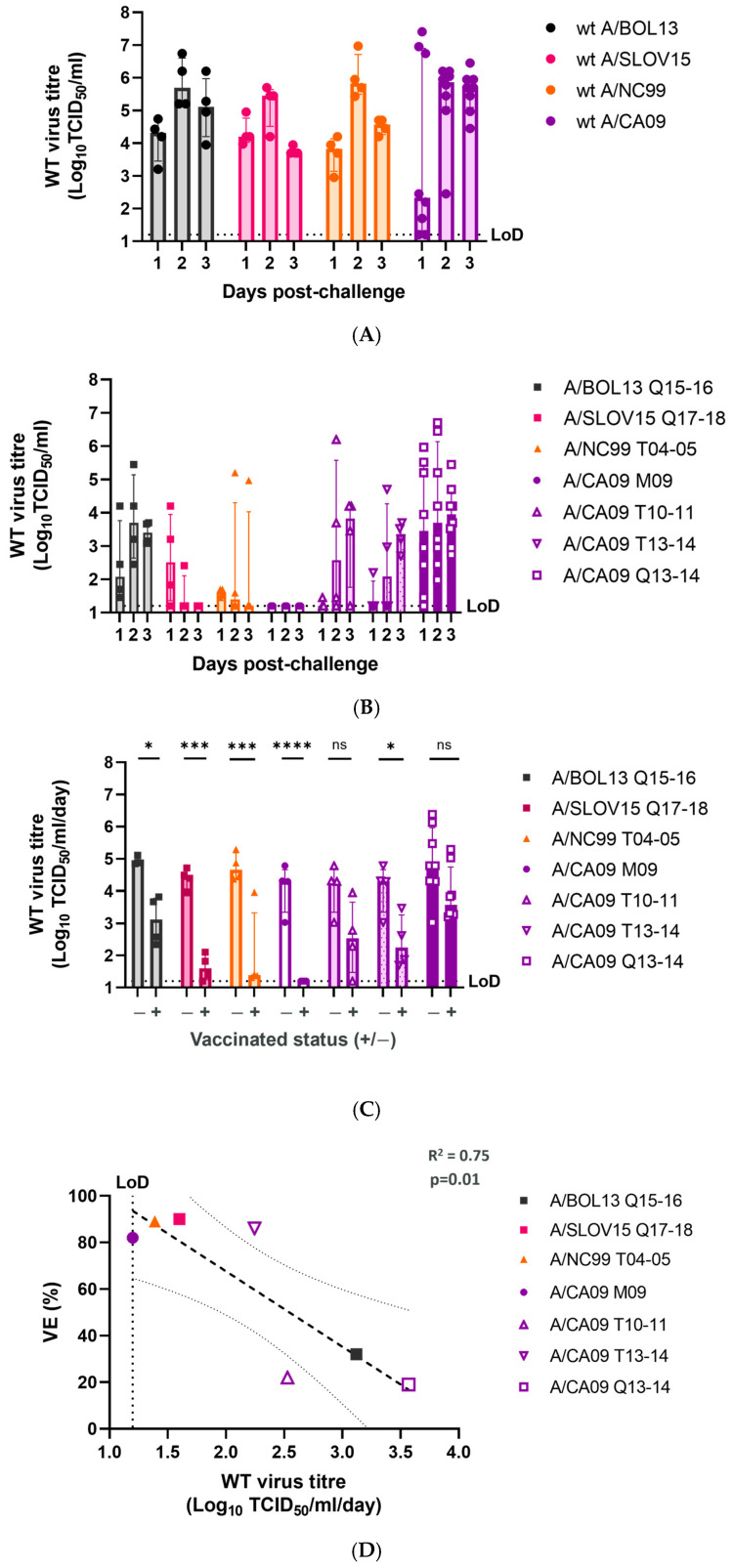
LAIV formulations with higher VE protect ferrets from *wt* virus shedding post-challenge. At d28 post-challenge, all ferrets were challenged with 5 Log_10_ FFU of *wt* virus homologous to the vaccinating H1N1 strain for that group. Shedding of *wt* virus was measured daily for 3 days by TCID_50_ assay. (**A**) Daily shedding of *wt* challenge viruses in unvaccinated animals. (**B**) Daily *wt* virus shedding in vaccinated groups. (**C**) Comparison of geometric mean *wt* virus shedding per day in vaccinated (+) and unvaccinated control (-) animals. Points represent individual animals, while columns and error bars show group median and interquartile range. Statistical significance of comparisons in C are indicated by horizontal lines and labelled as: ns *p* > 0.05; * *p* < 0.05; *** *p* < 0.001; **** *p* < 0.0001. (**D**) Correlation of geometric mean *wt* virus shedding per day with H1N1 VE by linear regression. Points show the group median for each formulation. The heavy dashed line shows the linear regression and the light dashed lines the 95% confidence intervals. Linear regression statistics (R^2^, *p* value) are shown. Limit of detection of the TCID_50_ assay is indicated by a dashed line (LoD) in all cases.

To determine the extent to which protection from *wt* challenge would correlate with H1N1 VE, all ferrets were challenged with 5 Log_10_ FFU of the *wt* virus homologous to the vaccinating H1N1 strain for that group. Nasal swabs or nasal washes were taken daily for three days post-challenge and the *wt* virus titre was measured by TCID_50_ assay. The daily shedding titres for the *wt* challenge viruses used: *wt* A/BOL13, *wt* A/SLOV15, *wt* A/NC99, and *wt* A/CA09, are shown in Figure 3A. All *wt* strains shed with similar kinetics, with group median shedding peaking at d2 post-challenge, between ~5–6 Log_10_ TCID_50_/mL.

Vaccinated ferrets shed *wt* virus to considerably different levels, in a formulation-dependent manner (Figure 3B). A/BOL13 Q15-16 and A/CA09 Q13-14 animals shed *wt* virus most consistently over 3 days. Conversely, the A/CA09 M09 group produced no detectable *wt* virus shedding.

To facilitate statistical comparison of *wt* shedding between vaccinated and unvaccinated groups, a shed-virus-per-day statistic was calculated (Figure 3C). This was taken as the geometric mean of daily virus titres for each animal, as published previously [13,14,22]. Comparison of vaccinated and unvaccinated animals in this way showed that A/SLOV15 Q17-18 (*p* = 0.0001), A/NC99 T04-05 (*p* = 0.0001), and A/CA09 M09 (*p* < 0.0001) produced the most pronounced reductions in *wt* shedding. A/BOL13 Q15-16 also produced a significant difference between vaccinated and unvaccinated animals. However, the group median shedding for A/BOL13 Q15-16 remained notably higher, at approximately 3 Log_10_ TCID_50_/mL/day. Unlike HAI GMT, the levels of *wt* shedding correlated significantly with the clinical VE values (Figure 3D: R^2^ = 0.75, *p* = 0.01).

**Figure 4:**
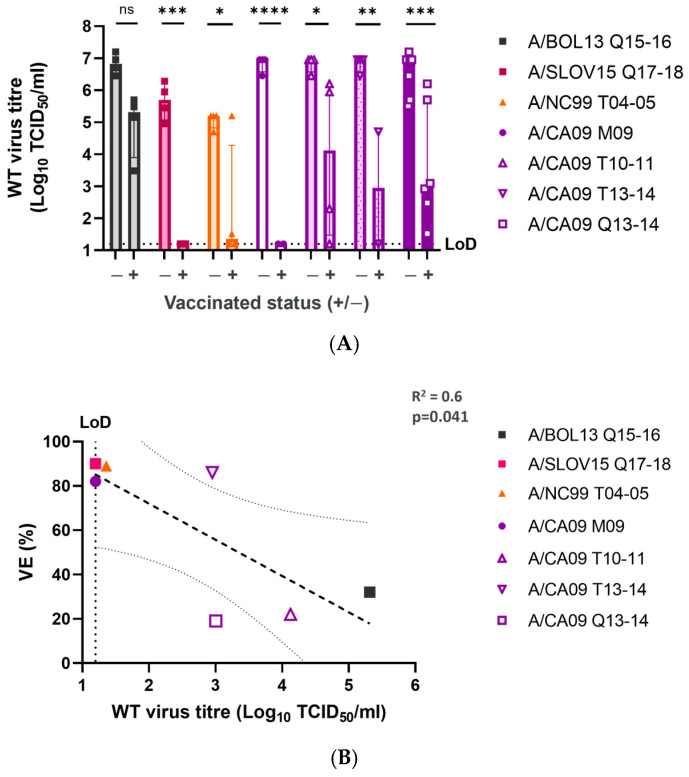
LAIV formulations with higher VE give greater reductions in *wt* virus load in nasal turbinate tissues. Following culling at d31 post-vaccination, NT tissues were removed from all study animals and the *wt* virus load was measured by TCID_50_ assay. (**A**) Comparison of *wt* challenge virus titres in vaccinated (+) and unvaccinated control (-) animals. Points represent individual animals, while columns and error bars show the group median and interquartile range. Statistical comparisons are indicated by horizontal lines and labelled as: ns *p* > 0.05; * *p* < 0.05; ** *p* < 0.01; *** *p* < 0.001; **** *p* < 0.0001.(**B**) Correlation of NT *wt* virus load and H1N1 VE (%) by linear regression. Points indicate group median for each formulation. The heavy dashed line shows the linear regression and the light dashed lines 95% the confidence intervals. Linear regression statistics (R^2^, *p* value) are shown. Limit of detection of the TCID_50_ assay is indicated by a dashed line (LoD).

In addition to *wt* virus shedding, the *wt* virus load in NT was measured for all animals at d3 post-challenge (Figure 4), by TCID_50_ assay. Levels of reduction in *wt* virus load in NT did not exactly mirror those seen for *wt* shedding. In fact, all vaccine formulations other than A/BOL13 Q15-16 significantly reduced the *wt* virus load in NT tissue (Figure 4A). However, the extent of that reduction varied considerably between groups, with A/SLOV15 Q17-18 (*p* = 0.0001), A/NC99 T04-05 (*p* = 0.026), and A/CA09 M09 (*p* < 0.0001) providing the greatest reduction in median *wt* virus titre. The viral load was highly variable in all other A/CA09 vaccine groups. Despite these variations, the *wt* virus load in NT tissues correlated significantly with clinical VE data (Figure 4B—R^2^ = 0.6, *p* = 0.041).

**Figure 5:**
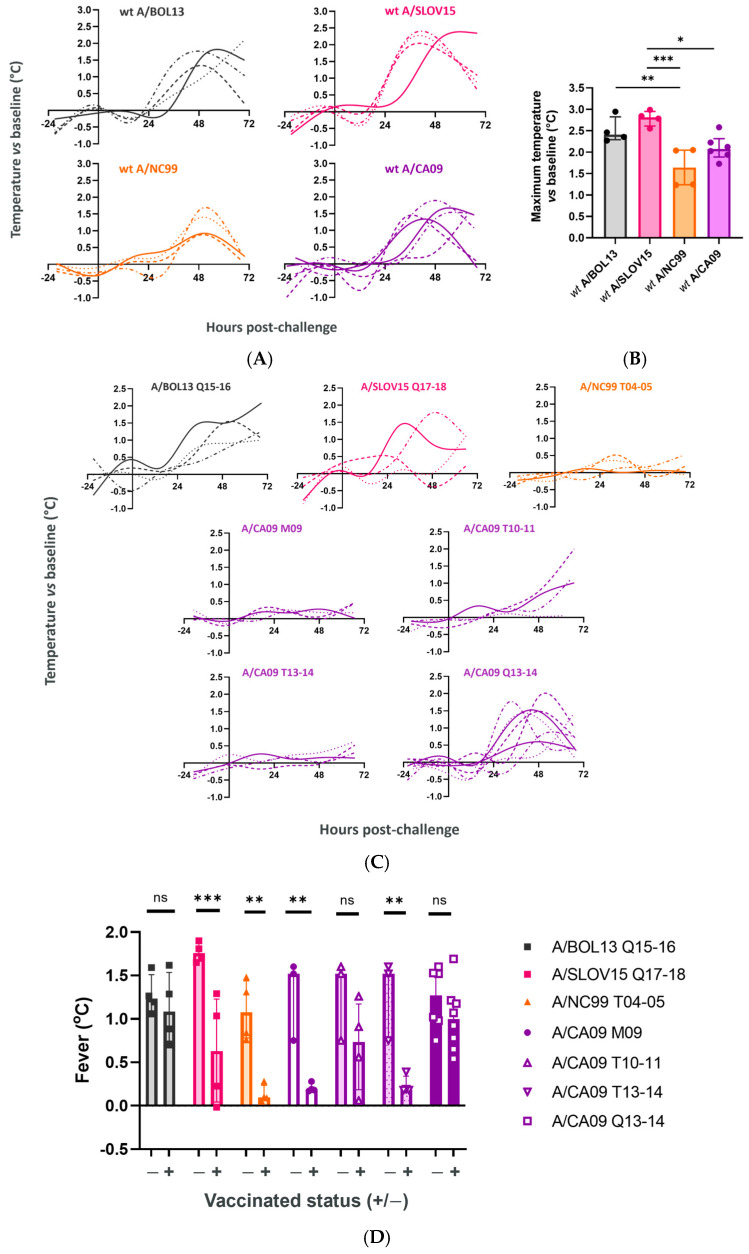

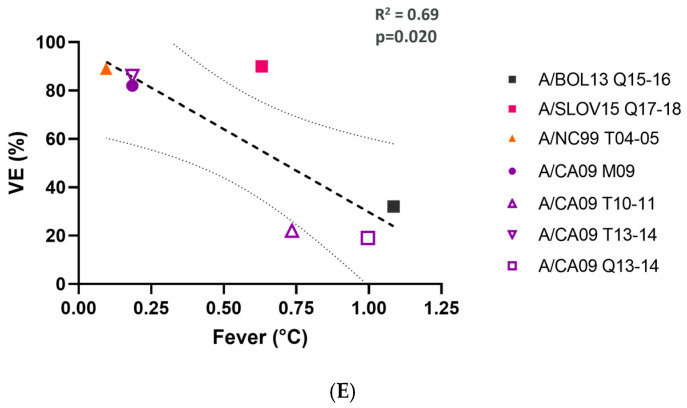
LAIV formulations with higher VE protect ferrets from fever. At d28 post-challenge, mock vaccinated and vaccinated ferrets were challenged intranasally with 5 Log_10_ FFU of *wt* virus homologous to the vaccinating H1N1 strain for that group. Generation of fever as a measure of influenza-like illness was monitored via implanted data loggers. Data points were taken at least hourly for the duration of the study, versus a pre-challenge average (baseline). ‘Fever’ for each animal was then calculated as the average temperature difference vs. baseline during a post-challenge window in which the unvaccinated control animal temperature was >1.5SD above baseline. (**A**) Change in body temperature vs. baseline in unvaccinated control animals, from 24 h pre-challenge to 72 h post-challenge (cull). *wt* challenge viruses are labelled (top of panel). (**B**) Maximum recorded temperature vs. baseline for *wt* viruses in unvaccinated animals. *wt* strains are labelled on the x-axis and the y-axis shows the single highest temperature vs. baseline recorded for each ferret. (**C**) Change in body temperature vs. baseline in vaccinated groups. Vaccine groups are labelled at the top of the panels. Individual lines are shown for each animal in a group, with spline curves fitted using 6 knots of smoothing. (**D**) Comparison of fever temperatures for vaccinated (+) and unvaccinated control (-) groups. In all panels, points represent individual animals while columns and error bars show the group median and interquartile range. The statistical significance of comparisons is indicated by horizontal lines and labelled as: ns *p* > 0.05; * *p* < 0.05; ** *p* < 0.01; *** *p* < 0.001. (**E**) Correlation of fever with H1N1 VE by linear regression. Points show the group median for each formulation. The heavy dashed line shows the linear regression and the light dashed lines the 95% confidence intervals. Linear regression statistics (R^2^, *p* value) are shown.

Finally, to assess the ability of LAIV H1N1 strains in different formulations to protect ferrets from influenza-like illness, fever development post-challenge was assessed. Smoothed spline plots of body temperature change relative to the pre-challenge baseline showed that all of the *wt* challenge viruses used in this study generated a consistent temperature increase post-challenge between animals (Figure 5A). However, there was variation observed in the magnitude of the temperature changes for different *wt* strains. Comparing the maximum recorded post-challenge temperature deviations vs. baseline for each strain (Figure 5B), *wt* A/NC99 (group median, 1.64 °C) produced a significantly lower temperature increase than either *wt* A/BOL13 (group median, 2.41 °C, *p* = 0.009) or *wt* A/SLOV15 (group median, 2.81 °C, *p* = 0.0008).

The temperature curves for vaccinated animals showed a variety of profiles (Figure 5C). A/BOL13 Q15-16- and A/CA09 Q13-14-vaccinated animals responded to challenge most similarly to unvaccinated controls. A/NC99 T04-05-, A/CA09 M09-, and A/CA09 T13-14-vaccinated animals exhibited minimal deviation from the baseline temperature. A/SLOV15 Q17-18 produced a partial phenotype, with 2/4 animals remaining close to the baseline temperature, and 2/4 animals producing unvaccinated-like temperature curves.

To facilitate statistical comparison, a single ‘fever’ temperature was calculated for each individual animal as previously described [13,14]. Briefly, a post-challenge ‘fever period’ was defined, during which unvaccinated control animals had temperatures >1.5SD over the pre-challenge baseline. For each study animal, the mean temperature difference vs. baseline during that window was then calculated and defined as ‘fever.’ Comparison of vaccinated and unvaccinated groups showed that A/BOL13 Q15-16 and A/CA09 Q13-14 produced no significant reduction in fever (Figure 5D). Conversely, A/SLOV15 Q17-18, A/NC99 T04-05, A/CA09 M09, and A/CA09 T13-14 all produced significant reductions in fever relative to unvaccinated controls.

Similarly to *wt* shedding, fever temperature produced a significant correlation with VE data (Figure 5E: R^2^ = 0.69, *p* = 0.02).

## 4. Discussion

The availability of clinically translatable animal models is critical for vaccine development. For influenza, the ferret is the preferred animal system for vaccine development, due to its physiological and immunological similarity to humans [24,25,26]. Here, we aimed to present a ferret efficacy model capable of reproducing H1N1 clinical data for LAIV. The model was derived from previously published work responding to the reduced VE of A/H1N1pdm09 strains used in the 2013–2014 and 2015–2016 influenza seasons [12,13,14]. Using a significantly reduced vaccine dose (4 Log_10_ FFU vs. 7 Log_10_ FFU clinical dose), it focused on protection endpoints that would be most representative of measurement of VE in the clinic.

Test-negative VE studies contributed the majority of clinical data referenced in this study [11,18,19], with randomised controlled trial efficacy data only available for the pre-2009 strain, A/NC99 [15]. Typically, the test-negative approach relies on a patient reporting with symptoms of influenza-like illness and then testing positive for influenza virus infection by nasal swab [27,28]. Here, measurement of both *wt* virus shedding in the ferret nose (R^2^ = 0.75) and fever development as a quantifiable measure of influenza-like illness (R^2^ = 0.69) resulted in significant correlations with clinical VE data for H1N1 LAIV. These data suggest that consideration of both endpoints can provide a reliable indication of a CVVs’ clinical performance. In addition, reductions in *wt* virus load in NT tissue correlated with clinical observations, providing a third, supportive endpoint. However, these data were inherently sparser due to being taken at a single timepoint. Importantly, taken together, these protection data showed that an optimised ferret efficacy model could provide an in vivo representation of clinical VE for H1N1 LAIV.

The ability to generate reproducible efficacy assessments was an important consideration in the development of this model. In vivo efficacy for the A/BOL13 Q15-16 and A/SLOV15 Q17-18 formulations had been described previously, [13,14]. Here, these studies were reproduced at a different institution with an independent ferret colony. Virus shedding was also sampled by nasal wash rather than nasal swab. Despite these methodological changes, A/BOL13 Q15-16 and A/SLOV15 Q17-18 produced very similar protection outcomes. Previously, at a 4 Log_10_ FFU QLAIV dose, A/BOL13 failed to provide significant reductions in either *wt* shedding or fever [13], while A/SLOV15 Q17-18 was able to confer significant protection against both endpoints, although in <100% of animals [14]. Here, a very similar profile was seen, identifying A/BOL13 Q15-16 as less protective than A/SLOV15 Q17-18. Again, A/SLOV15 provided significant protection but in less than 100% of animals, confirming the previous observations and underlining the reliability of the model.

One possible explanation for the partial protection seen for A/SLOV15 Q17-18 was the use of a cell-derived *wt* challenge virus (cell-*wt*), in place of egg-derived *wt* (egg-*wt*) viruses for all other groups. This was due to the inability of the egg-*wt* A/SLOV15 virus to produce a pathogenic infection (Figure S1). The major distinction between these two *wt* isolates was a Q223R (H1N1 numbering) egg-adaptation mutation in the HA protein. This mutation is common amongst recent egg-*wt* isolates and is implicated in creating an avian-like receptor binding profile [29,30]. Previously, in developing an A/SLOV15 LAIV CVV with enhanced hNEC replication, this mutation required reversion to the cell-*wt* sequence (223Q) [14]. Here, it is likely that this egg-adaptation of the egg-*wt* virus was responsible for reducing its replication in the ferret respiratory tract sufficiently to preclude it from inducing a fever response. Use of the A/SLOV15 cell-*wt* could then have provided a more stringent challenge than other *wt* viruses used. For example, while *wt* shedding for all challenge viruses was comparable, the cell-*wt* A/SLOV15 induced a significantly higher maximum post-challenge temperature than that of either pre-2009 *wt* A/NC99 or A/H1N1pmd09 *wt* A/CA09 (Figure 5B). Such differences may have influenced the precise level of protection observed for this strain and should be acknowledged. The frequent occurrence of this Q223R mutation in egg-derived A/H1N1pdm09 *wt* strains and its potential impact on the in vivo replication of *wt* and LAIV viruses alike will be an ongoing challenge, both for application of this efficacy model and for LAIV CVV development.

In addition to demonstrating the clinical translatability of the ferret efficacy model, this work aimed to understand the influence of changing vaccine composition on VE for a single H1N1 strain. To assess this, four A/CA09 vaccine formulations were investigated, with varying VE [11,18]. In each case, the A/CA09 LAIV strain was identical, with formulations only differing by H3N2 or B strain composition. The data presented show that vaccine composition was able to affect A/CA09 VE. A/CA09 M09 (82% VE, 95% CI, 14–96) provided the clearest protection from *wt* challenge in ferrets, while A/CA09 Q13-14 (19% VE, 95% CI, −18–44), assessed across two separate studies (8 animals total), gave no significant protection from either *wt* shedding or fever. This correlated with the clinical VE reported for each formulation [11].

The initial hypothesis proposed for the reduced VE of A/CA09 Q13-14 was thermal instability of the HA protein during shipping [31,32]. Subsequently, in vitro data were reported suggesting that thermal instability was not the primary cause of A/CA09 VE concerns, but rather reduced hNEC replication [12,33]. Here, all vaccine formulations were produced under R&D conditions and stored at −80 °C up to the point of thaw for administration to animals. This removed the possibility of HA protein exposure to environmental conditions impacting efficacy outcomes. These data showed that A/CA09 Q13-14 still produced low efficacy in ferrets in the absence of any heat exposure. Together with the high level of protection conferred by A/CA09 M09, this concurred with our previous conclusion that A/H1N1pdm09 LAIV strains with reduced replication in hNEC can suffer from inter-strain competition in vivo, reducing their efficacy [12,13]. This suggests that the optimisation of A/H1N1pdm09 LAIV strains for replication in hNEC, as described for A/SLOV15 [14], as well as the more recent A/Victoria/1/2020 [34], will continue to be critical for ongoing A/H1N1pdm09 LAIV CVV development.

In TLAIV formulations, A/CA09 produced a more intermediate phenotype. Both A/CA09 T10-11 (22% VE, 95% CI, 20–65) and A/CA09 T13-14 (86% VE, 95% CI, −11–98) formulations reduced *wt* shedding by a similar magnitude, although only A/CA09 T13-14 did so statistically significantly. By both the HAI titre and NT virus load, these formulations appeared similar. However, only A/CA09 T13-14 conferred protection against fever (median fever, 0.19 °C), with a distinct phenotype relative to A/CA09 T10-11 (median fever, 0.74 °C). A/CA09 T13-14 was also quantifiably more protective than A/CA09 Q13-14 in ferrets, the only difference being the addition of the B/BRIS08 strain in the A/CA09 Q13-14 vaccine. This suggested that A/CA09 may have been better able to compete in TLAIV than QLAIV. In the two TLAIV formulations tested, A/CA09 T10-11 and A/CA09 T13-14, both the H3N2 and B strain components were different (Figure 1A). This might indicate that A/CA09 was close to a hypothetical ‘competition threshold,’ capable of protection in TLAIV but sensitive to changes in the specific components of the vaccine. Further work will be required to fully understand the relative influence of non-A/H1N1pdm09 strain changes on A/H1N1pdm09 VE.

Although the measurement of *wt* shedding and fever currently provide the strongest indicators of VE in this model, continued optimisation might further increase the dynamic range and provide clearer differentiation between more intermediate phenotypes. For example, Marriott et al. reported that use of a low, physiologically relevant *wt* challenge dose (2 Log_10_ pfu/animal) was able to show greater antiviral activity for oseltamivir in ferrets [35]. Subsequently, the same approach revealed a reduction in disease severity due to LAIV-induced cross-reactive T-cells [36]. In our efficacy model, this could increase resolving power but would also tend to increase variability and so require larger study groups. This would need a balance of ethical study design with the potential for more nuanced outputs.

Another significant limitation of the current model in accurately translating VE data is the clinical data themselves. Test-negative VE studies are highly variable and may be conducted and analysed differently in different settings, with significant limitations relative to randomised controlled clinical trials [27,28,37]. Study size can be small, depending on influenza virus circulation and surveillance, meaning some of the VE estimates used as a basis for our assessment may be subject to variability. For example, while the A/CA09 T13-14 formulation gave a high VE estimate in Canada [18], the small size of the study resulted in wide confidence intervals (86% VE, 95% CI, −11–98). Similarly, for A/CA09 M09 VE data generated during the 2009 pandemic, the consolidation of three small studies in the USA resulted in a non-significant VE estimate of 79% (95% CI, −16–96) [11]. When the parameters of an individual study were adjusted to exclude only cases from ≤7-days post-vaccination, rather than ≤14 days post-vaccination, this resulted in a statistically significant VE estimate of 82% (95% CI, 14–96) [38], indicating the sensitivity of these data to the analysis method. Here, the decision was made to take VE point estimates at face value to enable study design and vaccine comparison. In addition, data from test-negative and randomised controlled trials were treated as equivalent, to allow hypothesis generation, despite their methodological differences. Indeed, the more intermediate phenotype seen for A/CA09 T13-14 could potentially be explained by the uncertainty of its VE estimate. Larger VE studies with more robust point estimates would be extremely beneficial to the continued understanding of VE and mechanism of action for LAIV.

Another key element of variability not accounted for in the current model is the complexity of the human immunological landscape. Clinical data, particularly those from test-negative VE studies, are derived from populations of varying age and infection history. Here, immunologically naïve ferrets of similar ages were used for simplicity and to isolate the virological properties of LAIV that might contribute to VE. The roles of immunological concepts such as repeated vaccination, pre-existing immunity, and imprinting in VE are subjects of ongoing discussion in the influenza field and remain unclear [37,39,40,41,42]. However, given LAIV is primarily used in paediatric populations, the naïve ferret model may be more appropriate for modelling this setting.

Despite the ability of this ferret model to reproduce H1N LAIV VE, there will be instances where the predictive capacity of any model can break down. Ongoing work will be required to correlate newly generated VE data with in vivo phenotypes and identify any inconsistencies. Ultimately, understanding the behaviour of a vaccine in its target host is of paramount importance. For LAIV, this could potentially be achieved by the use of human challenge models to describe LAIV-induced immunity and protection more fully. However, investigation of paediatric vaccination in this manner would be challenging.

During LAIV development, MLAIV CVVs are assessed for their antigenic match to WHO reference strains using post-infection antisera from ferrets. Here, serum immunogenicity in clinically relevant formulations, measured by HAI, offered minimal correlation with protection from challenge and clinical VE. This would explain the inability of standard pre-clinical immunogenicity assessments to predict the reduced VE of A/H1N1pdm09 LAIV strains between 2013 and 2016. It also suggests that, for LAIV, serum immune responses should not be relied on as an indicator of H1N1 VE in vivo. This concurs with historical clinical observations that serum antibodies are not the sole mediator of protection for LAIV and that seroconversion is not necessarily required for protection [4,43]. This is distinct from systemically delivered influenza vaccines, such as IIV, where serum neutralising antibodies against the HA protein are accepted as being the major correlate of protection [4,5,6]. Systemic neutralising antibody titres remain an important element of vaccine-induced immunity and are associated with protection from symptomatic disease for other pathogens, such as severe acute respiratory syndrome coronavirus 2 [44,45].

Due to its intranasal delivery and productive infection of the nasal epithelia, it is likely that the protective elements of the LAIV immune response are found at the mucosa rather than systemically. Interestingly, recent work by Thwaites et al. found that mucosal immune responses in LAIV vaccinees can be compartmentalised from systemic responses [46], potentially helping to explain the lack of correlation between serum antibody responses and protection from disease for LAIV.

LAIV has been shown to generate local B-cell and IgA antibody responses at the mucosa [46,47,48]. Their specific role in controlling influenza virus infection remains unclear. In addition, the possibility that mucosal antibody responses to influenza virus infection target viral antigens other than the HA protein remains relatively unexplored. For example, serum anti-NA antibodies have been implicated in protection from influenza disease [49], yet little is known about anti-NA antibodies at the mucosal surface, where the limitation of virus replication takes place. LAIV, utilising live, replication-competent viruses, induces anti-NA antibody responses [48]. It is possible that these could contribute to VE, independently of serum HA antibodies.

In addition to antibody responses, LAIV induces cross-reactive T-cells in respiratory tract tissues [50,51,52,53]. T-cells have been described as an important mechanism for the limitation of both influenza virus infection and disease in humans [54,55]. In the ferret model, LAIV-induced T-cell responses were shown to reduce the disease severity of heterosubtypic *wt* challenge [36]. Le Sage et al. also showed that *wt* influenza virus infection could provide protection from aerosol transmission of a heterosubtypic *wt* strain. While no mechanism was identified for this protective effect, it was found not to correlate with serum antibodies, making cross-reactive T-cell responses an appealing explanation [56].

Mucosal immunity to influenza and other respiratory viruses remains relatively poorly understood. Considerable work remains to address these knowledge gaps, both clinically and in vivo. While attempting to describe mucosal mechanisms of LAIV-induced protection was outside the scope of the studies described here, the availability of a model able to translate H1N1 LAIV VE data will prove invaluable to the investigation of LAIV mechanism of action and the role of alternative mediators of protection in vivo. One significant caveat to this is that the ferret has limitations as a model for mechanistic understanding of influenza immunology; notably a lack of analytical reagents when compared to more common systems, such as the mouse [57,58]. The generation of more comprehensive reagents for immunological characterisation in ferrets will be key to unlocking this model’s full potential.

## 5. Conclusions

In summary, the work presented demonstrates the ability of a ferret efficacy model, optimised for a low vaccine dose and clinically relevant endpoints, to reproduce clinical data for H1N1 LAIV. Using a greatly reduced vaccine dose and focusing on protection from challenge, in order to mirror real-world VE studies, it was shown that the ability to reduce both fever and *wt* virus shedding correlated strongly with clinical observations for a series of seven LAIV formulations. Conversely, serum antibody responses against the HA protein—the traditional pre-clinical measure of influenza vaccine immunogenicity—did not provide a strong indicator of clinical outcomes, explaining the lack of forewarning of reduced VE for A/H1N1pdm09 LAIV strains. It was also shown that varying vaccine composition can influence the VE of a single LAIV strain with reduced replication in hNEC, reiterating the importance of inter-strain competition for H1N1 LAIV. The availability of an animal model able to accurately reflect VE in humans will be invaluable for the future investigation of mechanism of action and vaccine virus optimisation for LAIV, particularly the exploration of mucosal mediators of protection. In broader terms, this also demonstrates the necessity of investing in the development of physiologically representative animal models for vaccine development.

## Data Availability

The original contributions presented in the study are included in the article/Supplementary Materials, and further inquiries can be directed to the corresponding author.

## Supplementary Materials

The following supporting information can be downloaded at https://www.mdpi.com/article/10.3390/vaccines12111275/s1. Table S1. Strain compositions of the 7 LAIV formulations investigated. Names of strains from each subtype across the 7 formulations described are shown. N/a indicates no strain included, in MLAIV and TLAIV formulations. Strain abbreviations used elsewhere are shown below the full strain name. Figure S1. Egg-derived *wt* A/SLOV15 replicated to reduced levels and failed to induce fever on ferret challenge, relative to cell-derived *wt* A/SLOV15. (A) Shedding of egg-derived *wt* A/SLOV15 (egg-*wt* A/SLOV15) and cell-derived wt A/SLOV15 (cell-*wt* A/SLOV15) following a 5 log_10_ FFU challenge dose. Virus titre in nasal washes taken daily for 3 days post-challenge were measured by TCID_50_ assay. (B) Geometric mean shedding per day was calculated, with statistical comparison shown. (C) Ferret body temperature relative to pre-challenge baseline following challenge. Spline curves with 6 knots of smoothing were fitted to hourly data points for each individual ferret. Individual animals are represented by lines with different patterns (4 animals per group. Turquoise lines = egg-*wt* A/SLOV15, pink lines = cell-*wt* A/SLOV15). (D) A single Fever temperature for each animal was calculated, with statistical comparison shown. The statistical significance of comparisons is indicated by horizontal lines and labelled as: ns *p* > 0.05; * *p* < 0.05; ** *p* < 0.01; *** *p* < 0.001; **** *p* < 0.0001.
