# Supplementary material for: An Optimised Live Attenuated Influenza Vaccine Ferret Efficacy Model Successfully Translates H1N1 Clinical Data
Source: Vaccines (Basel). 2024 Nov 13;12(11):1275. doi: 10.3390/vaccines12111275 (PMC11598904; doi:10.3390/vaccines12111275)
Supplement: Supplementary file 1 [file vaccines-12-01275-s001.zip › vaccines-3238289-supplementary.pdf]

Table S1. Strain compositions of the 7 LAIV formulations investigated. Names of strains from each subtype across the 7 formulations described are shown. N/a indicates no strain included, in MLAIV and TLAIV formulations. Strain abbreviations used elsewhere are shown below the full strain name.

Formulation name	H1N1	H3N2	B Victoria	B Yamagata
**A/BOL13 Q15-16**	A/Bolivia/559/2013 (A/BOL13)	A/Texas/50/2012 (A/TEX12)	B/Brisbane/60/2008 (B/BRIS08)	B/Phuket/3073/2013 (B/PHUK13)
**A/SLOV15 Q17-18**	A/Slovenia/2903/2015 (A/SLOV15)	A/New Caledonia/71/2014 (A/NC14)	B/Brisbane/60/2008 (B/BRIS08)	B/Phuket/3073/2013 (B/PHUK13)
**A/NC99 T04-05**	A/New Caledonia/20/1999 (A/NC99)	A/Wyoming/03/2003 (A/WY03)	n/a	B/Jilin/20/2003
**A/CA09 M09**	A/California/07/2009 (A/CA09)	n/a	n/a	n/a
**A/CA09 T10-11**	A/California/07/2009 (A/CA09)	A/Perth/16/2009 (A/PER09)	B/Brisbane/60/2008 (B/BRIS08)	n/a
**A/CA09 T13-14**	A/California/07/2009 (A/CA09)	A/Texas/50/2012 (A/TEX12)	n/a	B/Massachusetts/02/2012 (B/MASS12)
**A/CA09 Q13-14**	A/California/07/2009 (A/CA09)	A/Texas/50/2012 (A/TEX12)	B/Brisbane/60/2008 (B/BRIS08)	B/Massachusetts/02/2012 (B/MASS12)

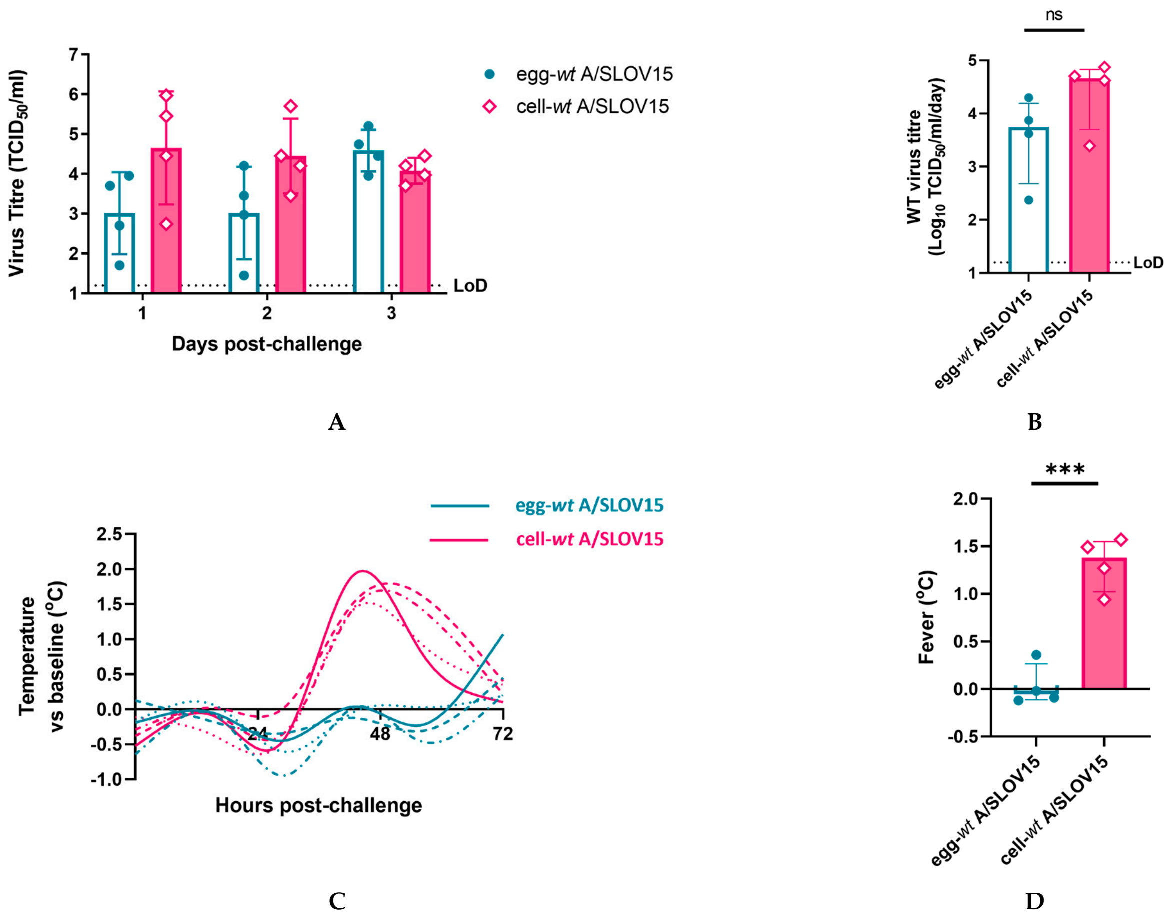

Figure S1. Egg-derived *wt* A/SLOV15 replicated to reduced levels and failed to induce fever on ferret challenge, relative to cell-derived *wt* A/SLOV15. **(A)** Shedding of egg-derived *wt* A/SLOV15 (egg-*wt* A/SLOV15) and cell-derived wt A/SLOV15 (cell-*wt* A/SLOV15) following a 5 $\log_{10}$ FFU challenge dose. Virus titre in nasal washes taken daily for 3 days post-challenge were measured by $TCID_{50}$ assay. (**B**) Geometric mean shedding per day was calculated, with statistical comparison shown. (**C**) Ferret body temperature relative to pre-challenge baseline following challenge. Spline curves with 6 knots of smoothing were fitted to hourly data points for each individual ferret. Individual animals are represented by lines with different patterns (4 animals per group. Turquoise lines = egg-*wt* A/SLOV15, pink lines = cell-*wt* A/SLOV15). (**D)** A single Fever temperature for each animal was calculated, with statistical comparison shown. The statistical significance of comparisons is indicated by horizontal lines and labelled as: ns $p > 0.05$; * $p < 0.05$; ** $p < 0.01$; *** $p < 0.001$; **** $p < 0.0001$.
